# Optimized expression of Peptidyl-prolyl cis/transisomerase cyclophilinB with prokaryotic toxicity from *Sporothrix globosa*

**DOI:** 10.1093/jimb/kuae017

**Published:** 2024-05-10

**Authors:** Ling Hu, Baicheng Deng, Rong Wu, Miaorong Zhan, Xuchu Hu, Huaiqiu Huang

**Affiliations:** Department of Dermatology and Venereology, The Third Affiliated Hospital of Sun Yat-Sen University, Guangzhou, Guangdong 510630, China; Department of Parasitology, Zhongshan School of Medicine, Sun Yat-sen University, Guangzhou, Guangdong 510030, China; Department of Dermatology and Venereology, The Third Affiliated Hospital of Sun Yat-Sen University, Guangzhou, Guangdong 510630, China; Department of Dermatology and Venereology, The Third Affiliated Hospital of Sun Yat-Sen University, Guangzhou, Guangdong 510630, China; Department of Parasitology, Zhongshan School of Medicine, Sun Yat-sen University, Guangzhou, Guangdong 510030, China; Department of Dermatology and Venereology, The Third Affiliated Hospital of Sun Yat-Sen University, Guangzhou, Guangdong 510630, China

**Keywords:** Cyclophilin B, *Sporotrix globosa*, Optimized expression

## Abstract

Cyclophilin B (CypB), a significant member of immunophilins family with peptidyl-prolyl *cis-trans* isomerase (PPIase) activity, is crucial for the growth and metabolism of prokaryotes and eukaryotes. *Sporothrix globosa* (*S. globosa*), a principal pathogen in the *Sporothrix* complex, causes sporotrichosis. Transcriptomic analysis identified the *cypB* gene as highly expressed in *S. globosa*. Our previous study demonstrated that the recombinant *Escherichia coli* strain containing *SgcypB* gene failed to produce sufficient product when it was induced to express the protein, implying the potential toxicity of recombinant protein to the bacterial host. Bioinformatics analysis revealed that *Sg*CypB contains transmembrane peptides within the 52 amino acid residues at the N-terminus and 21 amino acids near the C-terminus, and 18 amino acid residues within the cytoplasm. AlphaFold2 predicted a *Sg*CypB 3D structure in which there is an independent PPIase domain consisting of a spherical extracellular part. Hence, we chose to express the extracellular domain to yield high-level recombinant protein with PPIase activity. Finally, we successfully produced high-yield, truncated recombinant CypB protein from *S. globosa* (*Sg*trCypB) that retained characteristic PPIase activity without host bacterium toxicity. This study presents an alternative expression strategy for proteins toxic to prokaryotes, such as *Sg*CypB.

**One-Sentence Summary:**

The recombinant cyclophilin B protein of *Sporothrix globosa* was expressed successfully by retaining extracellular domain with peptidyl-prolyl *cis-trans* isomerase activity to avoid toxicity to the host bacterium.

## Graphical Abstract

**Graphical Abstract:**
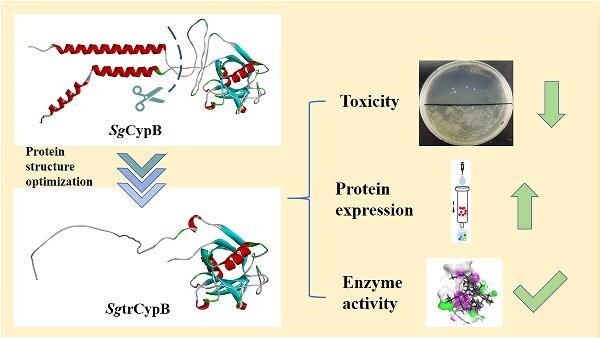

## Introduction

Cyclophilin B (CypB) belongs to the immunophilins family and has peptidyl-prolyl *cis-trans* isomerase (PPIase) activity, which catalyzes the *cis-trans* isomerization process of proline residues, widely existing in eukaryotes and prokaryotes (Wang & Heitman, 2005). Cyclophilins (Cyps) are intracellular protein targets of the immunosuppressive drug cyclosporin A (CsA), and the PPIase activity is strongly inhibited by CsA through binding PPIase active site (Swanson et al., 1992; Walsh et al., 1992). Fungal PPIases are widely involved in a range of biological processes, including virulence, growth, stress response, and metabolic regulation (Dimou et al., 2017; Singh et al., 2018). Studies on *Cryptococcus neoformans* showed that Cyps are pivotal in growth, mating, and virulence. Their absence or mutation diminishes the stress tolerance of fungi. (Wang et al., 2001). Infection experiments with *Beauveria bassiana* on *Plutella xylostella* larvae indicated that CypB upregulation is associated with fungal invasion and virulence (Collette & Lorenz, 2011). Given the conserved nature of Cyps across fungi, they may play analogous roles in pathogenesis, such as adhesion and invasion. (Chen et al., 2011). Recently, a nanoscale liquid chromatography coupled with tandem mass spectrometry approach was used in a research to provide yeast proteomic profiles of *Sporothrix*. Cyclophilin B was identified, showing a higher expression in *Sporothrix globosa* (*S. globosa*)*.* (Silva-Bailão et al., 2021)*.*

*Sporothrix* is an etiological agent of sporotrichosis, which is an implantation mycosis, mainly including *S. schenckii, S. brasiliensis*, and *S.globosa.* The most common clinical manifestations of this mycosis are acute or chronic cutaneous and lymphocutaneous lesions, occurring after contact with soil, sphagnum moss, thorny plants, and even certain animals like cats, preferably in tropical and subtropical regions (Chakrabarti et al., 2015; Nava-Pérez et al., 2022). Research on *Sporothrix* virulence has predominantly focused on *S. schenckii* and *S. brasiliensis*, noted for higher virulence than *S. globosa*. (Tamez-Castrellón et al., 2020). However, virulence factors associated with *S. globosa* were poorly described, among which more were melanin and the fungal cell wall components, such as polysaccharide, chitin, Gp70, Gp60, and enolase (Ruiz-Baca et al.,^ 2009^; Alba-Fierro et al., 2016; Félix-Contreras et al., 2020, Portuondo et al., 2019; Villalobos-Duno et al., 2021). Our previous bioinformatical analysis found CypB was a potential membrane protein, located in the surface of *S. globosa* (Zixian et al., 2023)*.* However, we were unable to obtain the full-length recombinant protein as the bacterium lysed upon induction. Hence, it was necessary to optimize the expression of *Sg*CypB.

Previous studies have shown that the prokaryotic expression of eukaryotic membrane proteins is a great challenge, leading to low yield and easy formation of inclusion bodies (He et al., 2014). The main reason is the membrane proteins are hydrophobic, resulting in a toxic effect on host cells (Kesidis et al., 2020). At the same time, a large amount of recombinant eukaryotic membrane proteins expressed on the host membrane can change its characteristics and cause toxicity to the host cells. Therefore, prokaryotic expression of membrane proteins mainly expresses the soluble region, not the full-length protein (Laage & Langosch, 2001). This is because of the differences between prokaryotic and eukaryotic membranes and the lack of cellular environment such as post-translational modification during expression (Bill et al., 2011). Despite these limitations, bacterial cells remain a popular choice for their cost-effectiveness, high yield potential, and the relative ease of genetic manipulation. (Kesidis et al., 2020). Meanwhile, to the best of our knowledge, little is known about the prokaryotic expression for full length of eukaryotic CypB protein, and that only a hydrophobic leader sequence was truncated in the prokaryotic expression of human CypB has been reported (Price et al., 1991). Consequently, we only retained the extracellular part of *Sg*CypB, with the aim of obtaining a large amount of recombinant protein with PPIase activity, as it is involved in the pathogenesis of sporotrichosis.

We are reporting the successful high-level expression of *Sg*CypB protein with the PPIase enzymatic activity in *Escherichia coli (E. coli)* BL21(DE3) cells using the pET30a(+) expression vector system. This approach represents a viable option for expressing proteins that are toxic to prokaryotic systems.

## Methods

### Source of *S. Globosa cypB* Gene Sequence

The whole genome sequence of *S. globosa* (ASM163044v1, GenBank assembly accession: GCA_001 630 445.1) was gained by retrieving the National Center for Biotechnology Information (NCBI). The CDS sequence of *SgcypB* gene was obtained via sequence alignment with the CDS sequence of the *S. schenckii* cyclophilin B gene (*SscypB*) (NCBI Reference sequence: XM_016 733 058.1) and the whole genome sequence of *S. globosa*.

### Structure and Function Analysis By Bioinformatics

DeepTMHMM (https://dtu.biolib.com/DeepTMHMM) was utilized to predict the transmembrane regions of the putative full-length *Sg*CypB. AlphaFold2 (v2.0) (https://github.com/deepmind/alphafold) were employed to predict the structure. The highest ranking structural model of *Sg*CypB and *Sg*trCypB were selected as the receptors for molecular docking. The structure of CsA was the ligand for molecular docking, downloaded directly from PubChem (https://pubchem.ncbi) (PubChem ID:5 284 373). The potential natural binding sites were predicted using Discovery Studio 2019. LibDock pattern was used for high-precision docking. Entrusting receptors and ligands with CHARMm forcefields to estimate the binding free energy. GROMACS 2023 was used to calculate the average root mean square deviation (RMSD) and radius of gyration (Rg) of each docking result. The open-source version of PyMol (https://sourceforge.net/projects/pymol/) was applied to prepare the images.

### The Synthesis of the Gene Encoding the Extracellular Part of *Sg*CypB Protein and the Construction of the Recombinant Plasmid

Through our previous analysis of the characterization of *Sg*CypB, according to the amino acid sequence of the extracellular part, we chose the codons preferred by *E.coli* to artificially synthesize the DNA fragment *Sg*trCypB encoding 218 amino acid residues. The DNA fragment was synthesized and cloned into pET30a(+) vector through Nde I and Xho I sites by Tsingke (Guangzhou, China). The His-tag at C-terminal of this vector was retained for purification and identification. The recombinant expression vector pET30a-*Sg*trCypB was transformed into host bacterium *E. coli* strain BL21(DE3).

### Prokaryotic Expression of *Sg*trCypB

The recombinant *E. coli* BL21(DE3) was grown in Luria–Bertani (LB) broth with kanamycin until the OD_600_ reached 0.5. Subsequently, 0.5 mM isopropyl β-d-1-thiogalactopyranoside (IPTG) was added to induce expression of the *Sg*trCypB. The mixture was incubated in a thermostatic shaker at 200 rpm and 37°C for 2 hr, 4 hr, and 6 hr, then 12% sodium dodecyl sulfate-polyacrylamide gel electrophoresis (SDS-PAGE) were performed to identify the prokaryotic expression preliminarily and determine the optimum induction time. The number of viable bacterium carrying *Sg*CypB or *Sg*trCypB was assessed by determining the number of colony-forming units (CFUs) from the LB broth of different induction time, which was done by plating an appropriate dilution of the culture medium on LB agar plates. Meanwhile, the OD_600_ of different induction time was determined by spectrophotometer.

### Purification of Recombinant *Sg*trCypB

We then chose the optimum induction time to induce expression of the *Sg*trCypB in the 1 L of fresh LB medium containing 50 μg/mL kanamycin. The bacteria were then harvested and disrupted by sonication. The cells were lysed and then centrifuged to separate the supernatant and pellet. The SDS-PAGE method was used to identify the location of the recombinant protein. The supernatant filtered through a 0.45 µm pore membrane, and the Ni NTA Beads, which was purchased from Changzhou Smart-Lifesciences Biotechnology Co., Ltd (Changzhou, China), was utilized to purify the fusion protein in the supernatant according to the operating instructions. The recombinant proteins were dialyzed in phosphate-buffered saline for 18 hr at 4°C. Protein concentrations were measured using BCA assay method in accordance with the manufacturer's instructions.

### Western Blot Analysis

To identify the *Sg*trCypB, 12.5% SDS-PAGE and western blot analysis were performed. Protein samples were transferred to a polyvinylidene difluoride membrane (Merck, Germany) via electroblotting. The membrane was then blocked with 5%(w/v) skimmed milk powder in TBST (Tris Buffered Saline with Tween) for 1 hr and subsequently incubated with 1:1000 dilution rabbit anti-His antibody at 4°C overnight. After being washed with TBST three times, the membrane was incubated with 1:5000 dilution HRP-conjugated goat anti-rabbit IgG antibody at room temperature for 1 hr.

### PPIase Activity Assay

Peptidyl-prolyl *cis-trans* isomerase activity of the *Sg*trCypB was determined with an α-chymotrypsin-coupled PPIase assay (Fischer et al., 1992). A total mixture of 1 mL (100 μM N-succinyl-Ala-Ala-Pro-Phe-p-nitroanilide, 35 nM HEPES, 10 μM α-chymotrypsin, and *Sg*trCypB) was utilized, while double steam water was used to take the place of the *Sg*trCypB as the negative control. The reaction system (without α-chymotrypsin) was pre-cooled to 15°C. After adding α-chymotrypsin to the system, we used a spectrophotometer to determine the enzyme activity by continuously monitoring the change in absorbance at 390 nm.

### Statistical Analyses

The experiments were repeated at least three times. Data were presented as means ± standard deviations (mean ± SD) and analyzed using unpaired *t*-test or Mann–Whitney *U* test with GraphPad Prism 8.4.0 and SPSS 21.0 software. A *p*-value less than 0.05 was regarded as statistically significant.

## Results

### The Topological Structure of *Sg*CypB and Optimized Encoding Sequence

**Fig. 1.**
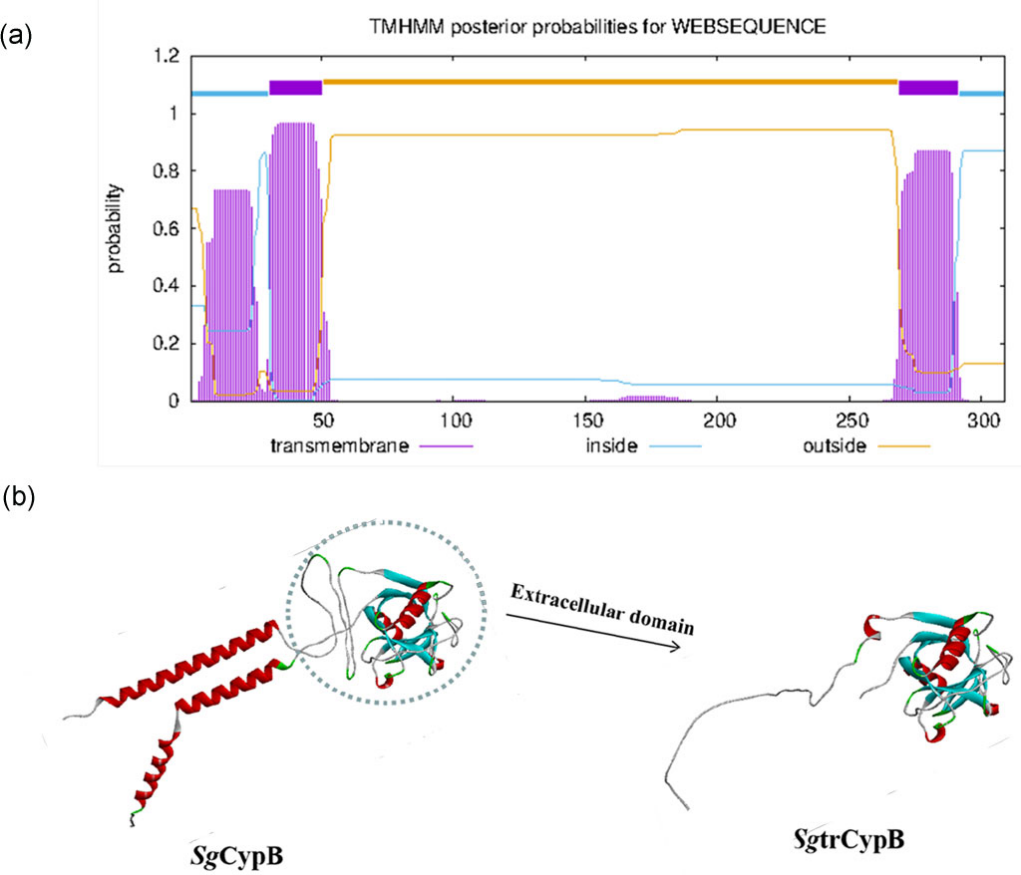
Prediction of transmembrane region of *Sg*CypB and 3D structure of *Sg*CypB and *Sg*trCypB. (a) From prediction of transmembrane region, retaining the sequence of outside was the *Sg*trCypB. (b) In the 3D structure of *Sg*CypB, the dashed area was the *Sg*trCypB.

The gene sequence was 930 bp long and encoded a putative protein containing 309 amino acids. (Supplementary Fig. S1). A 52-amino acid and 21-amino acid transmembrane regions located in 1–52aa and 271–291aa, respectively, predicted by DeepTMHMM (Fig. 1). Cyclophilin-type PPIase domain located in 63–220aa, with a signature pattern in 104–121aa, so the extracellular segment included all important binding and catalytic sites of PPIase. Then, to reduce the length and complexity of encoding sequence, we chose to truncate the transmembrane as well as intracellular regions and retain the extracellular domain, finally the sequence of *Sg*trCypB was determined, and the 3D structure of *Sg*CypB and *Sg*trCypB were illustrated in Fig. 1b. The transmembrane segments consisted of two α-helices located in the N and C terminal. The extracellular domain consisted of seven segments of β-lamination and two segments of α-helix forming a classical stable barrel structure with an inner hydrophobic and outer hydrophilic structure (Supplementary Fig. S2).

### Docking of CsA to *Sg*trCypB Compared With *Sg*CypB

Discovery Studio 2019 predicted that *Sg*trCypB and *Sg*CypB contain six and seven potential active pockets, respectively, but only the apical active pocket of the extracellular domain can stably docking with CsA, which is a natural CsA binding site based on previous amino acid sequence analysis (Zixian et al., 2023). Of the 50 docking results generated for each protein, the docking result with the highest score (LibDock score) was selected to display 2D and 3D interactions. Including hydrogen bond donor/acceptor and aromatic edge/face, the results were shown in Supplementary Fig. S3. The interactions between CsA and *Sg*trCypB or *Sg*CypB were dominated by the hydrophobic interactions between alkyl groups. Cyclosporin A also formed two conventional hydrogen bonds with Arg and Ala, and two hydrocarbon bonds with Pro and Thr. In order to evaluate the stability, complexes with binding energy less than 0 kcal/mol in LibDock docking results were selected to calculate the average RMSD and Rg values.

**Fig. 2.**
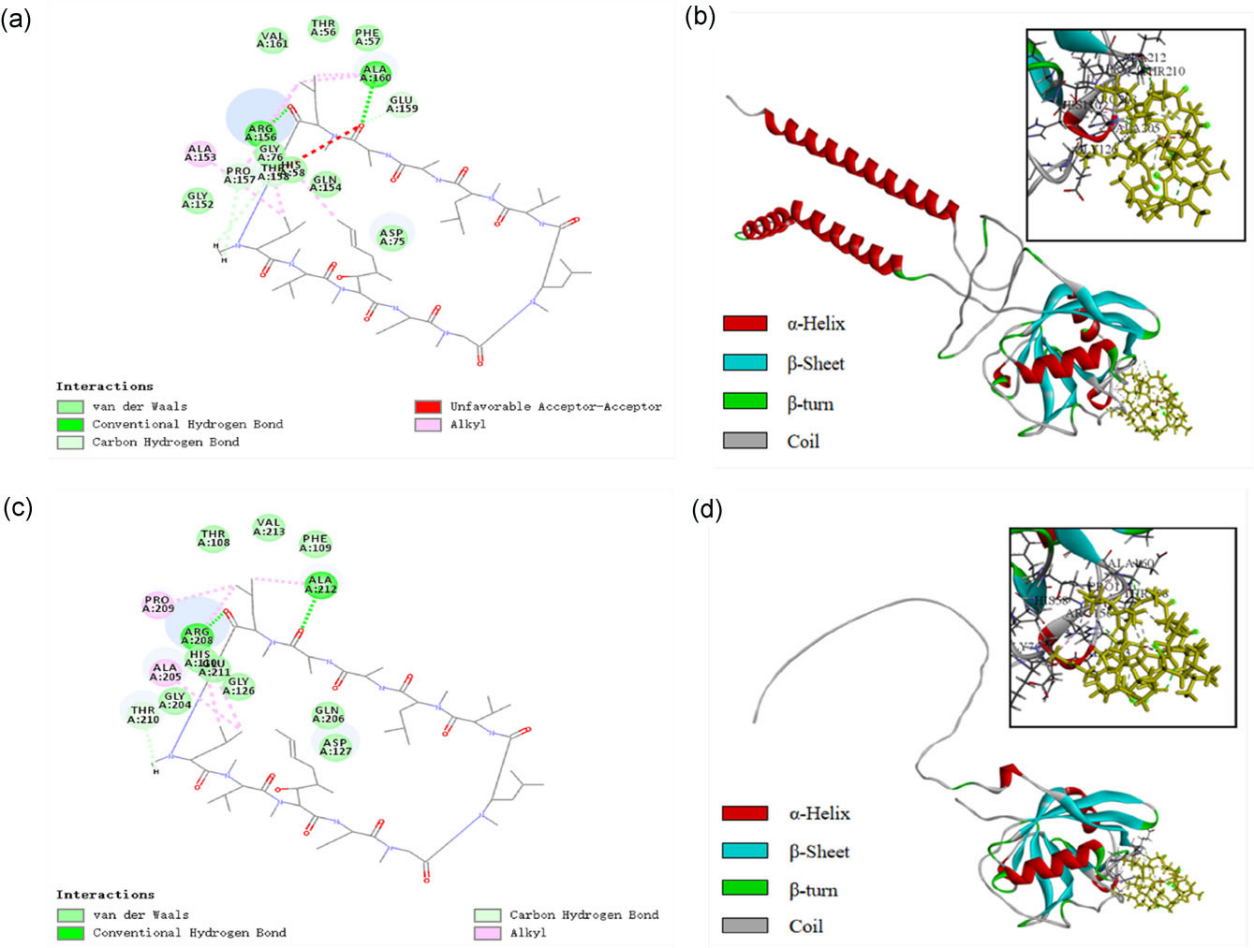
Molecular docking of CsA to *Sg*CypB and *Sg*trCypB. (a) The 2D representations of *Sg*CypB-CsA interactions. (b) The 3D representations of *Sg*CypB-CsA interactions. (c) The 2D representations of *Sg*trCypB-CsA interactions. (d) The 3D representations of *Sg*trCypB-CsA interactions.

Docking results showed that the amino acids of *Sg*CypB residues with strong interaction with ligands were Ala205, Arg208, Pro209, Thr210, Ala212, His110, and Gly126. (Binding energy: −3.39 kcal/mol, LibDock Score: 101.013, RMSD = 1.17 nm, Rg = 2.42 nm) (Fig. 2a–b). These amino acids were present in the active pocket of proline *cis-trans* isomerase. Compared with the docking of *Sg*trCypB, the amino acids of which were Ala153, Arg156, Pro157, Thr158, Ala160, His58, and Gly76, (Binding energy: −3.81 kcal/mol, LibDock Score: 103.320, RMSD = 1.26 nm, Rg = 1.87 nm) (Fig. 2c–d), we found the removal of those segments may not affect the PPIase activity. However, it may produce multiple configurations of the longer random coil segment at the C-terminus.

### The Comparison of Prokaryotic Expression Between *Sg*CypB and *Sg*trCypB in Different Induction Time By SDS-PAGE

**Fig. 3.**
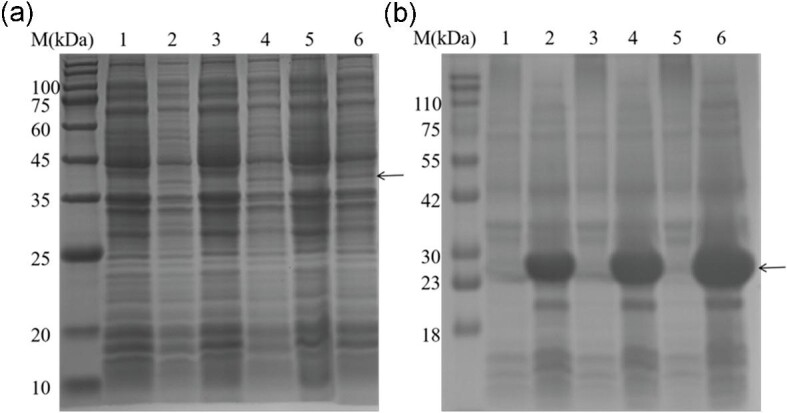
Expression of *Sg*CypB and *Sg*trCypB protein in different induction time analyzed by SDS-PAGE. (a) Expression of *Sg*CypB protein when induced by 0.5 mM IPTG in 2 hr, 4 hr, and 6 hr in SDS-PAGE. Lane 2h-, 4h-, 6h-: pET-30(a)-*Sg*CypB transformant without IPTG induction at the same time of induction for 2 h, 4 h, and 6 h. Lane 2h+, 4h+, 6h+ : pET-30(a)-*Sg*CypB transformant with IPTG induction for 2 hr, 4 hr, and 6 hr (expression of recombinant protein shown by the arrow). (b) Expression of *Sg*trCypB when induced by 0.5 mM IPTG in 2 hr, 4 hr, and 6 hr in SDS-PAGE. Lane 2h-, 4h-, 6h-: pET-30(a)-*Sg*trCypB transformant without IPTG induction at the same time of induction for 2 hr, 4 hr, and 6 hr. Lane 2h+, 4h+, 6h+ : pET-30(a)-*Sg*trCypB transformant with IPTG induction for 2 hr, 4 hr, and 6 hr (expression of recombinant protein shown by the arrow).

**Fig. 4.**
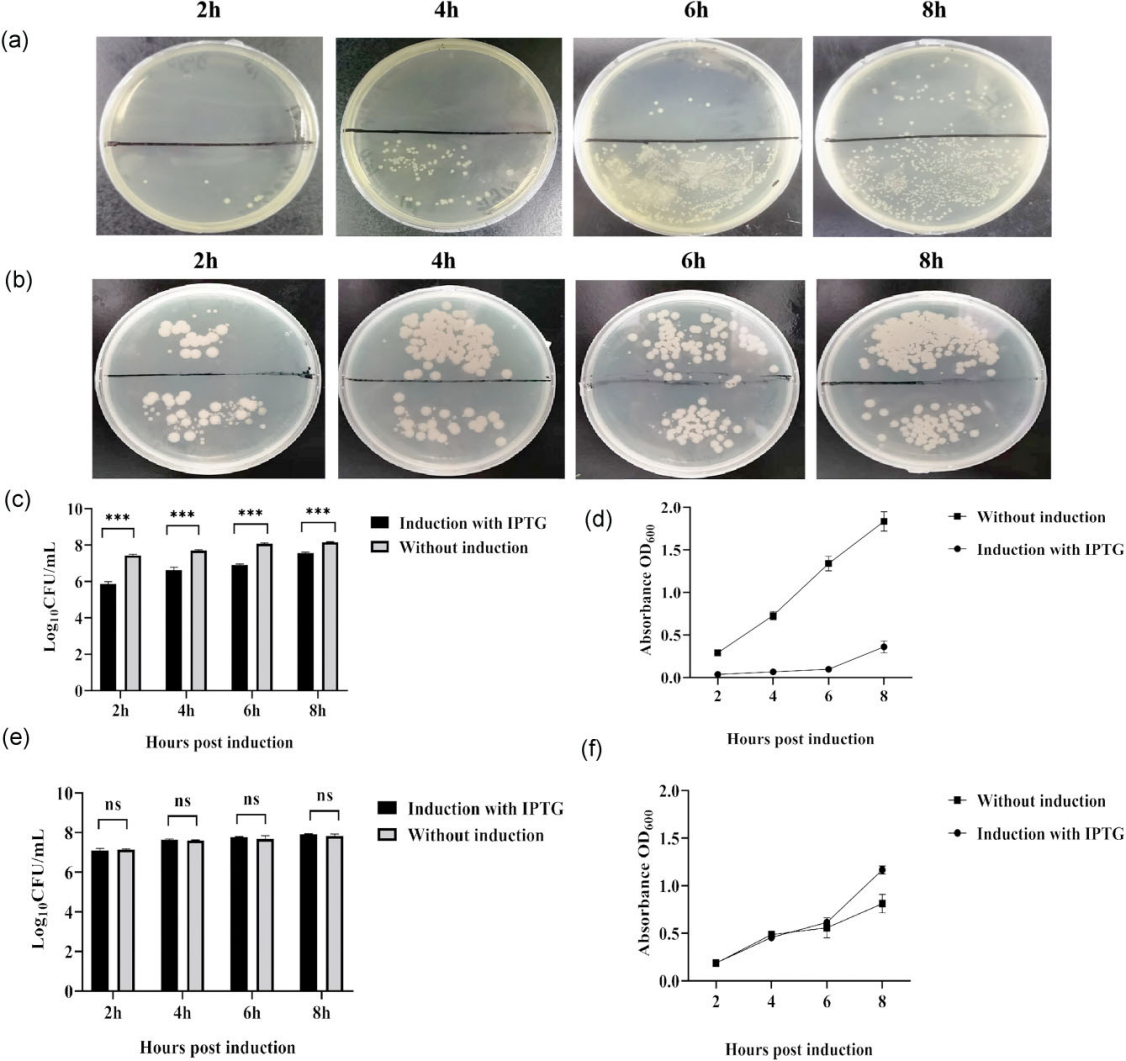
The colony-forming units of host bacteria when *Sg*CypB and *Sg*trCypB protein were expressed in different induction time. (a) The CFUs of host cells when pET-30(a)-*Sg*CypB transformant induced with IPTG for 2 hr, 4 hr, 6 hr, and 8 hr showed above the black line of plate, and The CFUs of pET-30(a)-*Sg*CypB transformant without induction at the same time were below the black line. (b) The CFUs of host cells when pET-30(a)-*Sg*trCypB transformant induced with IPTG for 2 hr, 4 hr, 6 hr, and 8 hr showed above the black line of plate, and The CFUs of pET-30(a)-*Sg*trCypB transformant without induction at the same time were below the black line. (c) Log _10_CFU/mL of pET-30(a)-*Sg*CypB transformant induced with IPTG for 2 hr, 4 hr, 6 hr, and 8 hr. *** refers to significant difference (*P *< 0.001). (d) OD_600_ of pET-30(a)-*Sg*CypB transformant induced with IPTG for 2 hr, 4 hr, 6 hr, and 8 hr. (*P *< 0.001).(e) Log _10_CFU/mL of pET-30(a)-*Sg*CypB transformant induced with IPTG for 2 hr, 4 hr, 6 hr, and 8 hr. ns refers to non-significant difference (*P* > 0.05). (F) OD_600_ of pET-30(a)-*Sg*CypB transformant induced with IPTG for 2 hr, 4 hr, 6 hr, and 8 hr (*P* > 0.05).

To preliminarily identify the expression of the recombinant protein, we performed SDS-PAGE with bacteria pellet at different induction times (Fig. 3). The result showed that the yield of *Sg*CypB was lower than *Sg*trCypB. Meanwhile, we found that the culture medium became clear and the number of host bacteria was less when the *Sg*CypB protein was expressed (*p* < 0.001), while the growth of host cells was unaffected when *Sg*trCypB protein was expressed (*p* > 0.05). (Fig. 4). These indicated that prokaryotic expression of full-length membrane protein *Sg*CypB produced toxicity to the host bacteria, resulting in the low yields of recombinant protein, which could be avoided by removing the trans-membrane domains.

### Expression, Purification, and Identification of *Sg*trCypB and the PPIase Assay of Recombinant *Sg*trCypB Using Double Steam Water As Negative control.

**Fig. 5.**
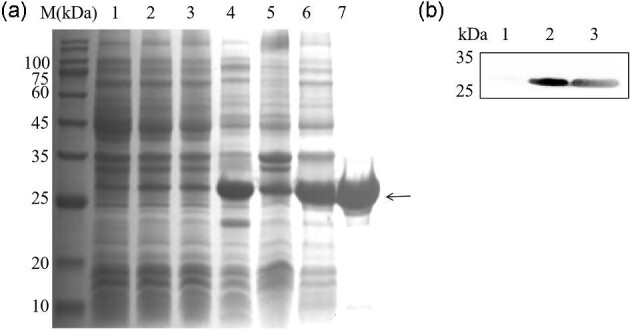
Expression and purification of r*Sg*trCypB protein analyzed by SDS-PAGE and Western blot analysis. (a) Expression and purification of r*Sg*trCypB protein analyzed by SDS-PAGE Lane M, protein marker; lane 1, pET-30(a) transformant without IPTG induction; lane 2, pET- 30(a) transformant with IPTG induction; lane 3, pET-30(a)-*Sg*trCypB transformant without IPTG induction; lane 4, pET-30(a)-*Sg*trCypB transformant with IPTG induction; lane 5, the lysate of recombinant bacteria with IPTG induction; lane 6, the supernatant of the lysate of recombinant bacteria with IPTG induction; lane 7, purified *Sg*trCypB protein (as pointed by the arrow). (b)Western blot analysis of *Sg*trCypB protein expression. lane 1, pET-30(a)-*Sg*trCypB transformant without IPTG induction; lane 2, pET-30(a)-*Sg*trCypB transformant with IPTG induction; lane 3, purified *Sg*trCypB protein.

The *Sg*trCypB was expressed as a fusion protein with a His-tag after being induced by IPTG. The recombinant protein was mainly in the supernatant of the lysate of the host bacteria, and the purified recombinant protein demonstrated a band in 12.5% SDS-PAGE with an approximate 25 kDa molecular weight. (Fig. 5a). At the same time, western blot analysis of *Sg*trCypB was conducted with an anti-His tag antibody. The negative control lane, namely pET30a-*Sg*trCypB transformed *E. coli* BL21(DE3) without IPTG induction, presenting no immunoreactive band. However, the lysate of the recombinant bacteria with IPTG induction and purified recombinant protein, displayed high immunoreactivity. (Fig. 5b).

**Fig. 6.**
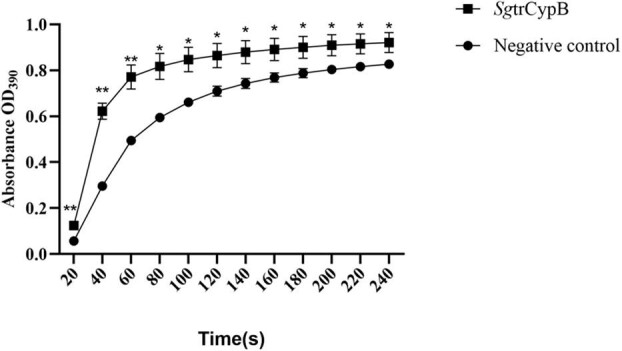
Peptidyl-prolyl cis-trans isomerase activity test of *Sg*trCypB with chymotrypsin-coupled assay using double steam water as negative control. * refers to significant difference (*P *< 0.05), ** refers to significant difference (*P *< 0.01), *** refers to significant difference (*P *< 0.001).

To verify whether the *Sg*trCypB protein retained PPIase activity, the purified *Sg*trCypB was tested with an α-chymotrypsin-coupled PPIase assay. As a result, the data demonstrated that there existed *cis-*to*-trans* conversion in the group of *Sg*trCypB protein compared with the negative control of double steam water, representing the enzymatic activity (Fig. 6).

## Discussion

In this study, we report a strategy to achieve high-level expression of *Sg*trCypB in *E. coli* cells, only retaining the vital extracelluar domain, thus reducing the size and structural complexity of recombinant protein while preserving enzyme activity. Homology modeling and molecular docking results predicted that *Sg*trCypB retained the active site in its 3D structure and that CsA could still bind to it. Additionally, we successfully obtained soluble and high-level *Sg*trCypB proteins that were identified specifically by anti-His antibody and the recombinant *Sg*trCypB maintained PPIase activity proved by PPIase assays.

We attempted to express the entire length of *Sg*CypB in *E. coli* BL21(DE3) cells. However, the quantity of recombinant protein obtained was insufficient and impure to conduct further research, despite using a gene sequence that was synthesized with *E. coli* preferred codons. During the process of induction, we observed that the culture medium became clear and the number of colonies decreased. This may be due to the death of host cells, which is consistent with other research on the prokaryotic expression of eukaryotic membrane protein (Grisshammer, 2006; Wagner et al., 2007). These studies suggest that producing membrane proteins in the cytoplasmic membrane may be toxic to *E. coli*. This is likely because a large amount of eukaryotic membrane protein needs to be expressed and inserted into the membrane of host cell, which can alter the metabolism or cytomembrane structure of *E. coli*. However, as an important member of immunophilin family, CypB plays a role of molecular chaperon and may not disturb biological processes in the host bacterium, so it may destroy the cytoplasm membrane and cell wall.

Human CypB prokaryotic expression studies have excluded the initial 25 amino acids due to a non-conserved hydrophobic leader sequence (Price et al., 1991). The process of fully expressing eukaryotic CypB in *E. coli* posed a significant challenge. In the specific case of *Sg*CypB, the transmembrane regions located in 1–52aa and 271–291aa were also hydrophobic, leading to a low yield and impure protein from *E. coli*. Little hydrophobic amino acid identified with tandem mass spectrometry (LC-MS/MS) in the recombinant *Sg*CypB protein proved the prokaryotic expression was a difficult task (Zixian et al., 2023). Optimizing expression in *E. coli* BL21(DE3) cells was necessary to obtain more proteins due to its cost-effectiveness, convenience, ease of operation, and time-saving benefits for prokaryotic expression. For the purpose of studying the enzymatic activity and function of the extracellular domain, we removed the sequence of transmembrane regions, including the following intracellular peptide which were not necessary. We predicted that the key residues in the *Sg*CypB protein were consistent with the *Sg*trCypB protein. This suggests that *Sg*trCypB protein is capable of binding to CsA and retaining its PPIase activity. As a result, the smaller size and simpler protein structure of *Sg*CypB protein had permitted its effective expression in prokaryotic expression systems, and *Sg*trCypB protein with PPIase activity was expressed successfully at a high level in *E. coli* cells.

It has been reported that human cyclooxygenase-2 (COX-2) protein was truncated to be expressed in *E. coli* BL21(DE3) cells. The truncated protein (trCOX-2) also maintained its enzyme activity. This study demonstrates the feasibility of expressing only the functional domain of a eukaryotic membrane protein in a prokaryotic expression system (Liao et al., 2017). Another approach was to express the extracellular domain of human programmed cell death 1 ligand 1 (PD-L1) in prokaryotes and investigate its function in order to identify potential drugs or antibodies. (Kalim et al., 2017). However, the human trCOX-2 protein and PD-L1 extracellular domain were mostly expressed as inclusion bodies, different from the *Sg*trCypB protein in our study, retaining natural conformation and biological function, which might result from the different molecular structure. In this study, PPIase activity of the recombinant protein was tested upon Sue-Ala-Xaa-Pro-Phe-4-nitroanilide, which is the most commonly used substrate of isomer-specific proteolysis using chymotrypsin as the protease. As shown in previous study, the PPIases of Cyps have been studied using tri- and tetrapeptide substrates and the refolding of denatured proteins. Chymotrypsin shows specificity for the trans-A-P amide conformer which it cleaves to produce the chromophore p-nitroaniline in a coupled assay, reflecting the PPIase activity (Fischer et al., 1992; Walsh et al., 1992).

However, we did not achieve high-level and pure yields of full-length *Sg*CypB. Future studies may explore yeast expression systems for full-length protein conformation and functional studies. This research did not compare PPIases between *Sg*trCypB and other Cyps, a topic for future research, focusing instead on assessing the enzymatic activity of optimized protein.

In conclusion, our approach provides a method to produce substantial quantities of functional *Sg*trCypB via prokaryotic expression, useful for studies requiring only the functional domain. Furthermore, *Sg*trCypB could be instrumental in developing CypB inhibitors. This strategy offers a valuable blueprint for expressing CypB proteins from eukaryotic organisms in prokaryotic systems and a method to optimize prokaryotic expression of eukaryotic membrane proteins, minimizing cytotoxicity, and achieving overexpression in *E. coli*.

## Supplementary Material

kuae017_Supplemental_Figures

## Data Availability

The data underlying this article are available in the article and in its online supplementary material.
