# Supplementary figures and images for: Optimized expression of Peptidyl-prolyl cis/transisomerase cyclophilinB with prokaryotic toxicity from Sporothrix globosa
Source: J Ind Microbiol Biotechnol. 2024 May 10;51:kuae017. doi: 10.1093/jimb/kuae017 (PMC11104532; doi:10.1093/jimb/kuae017)

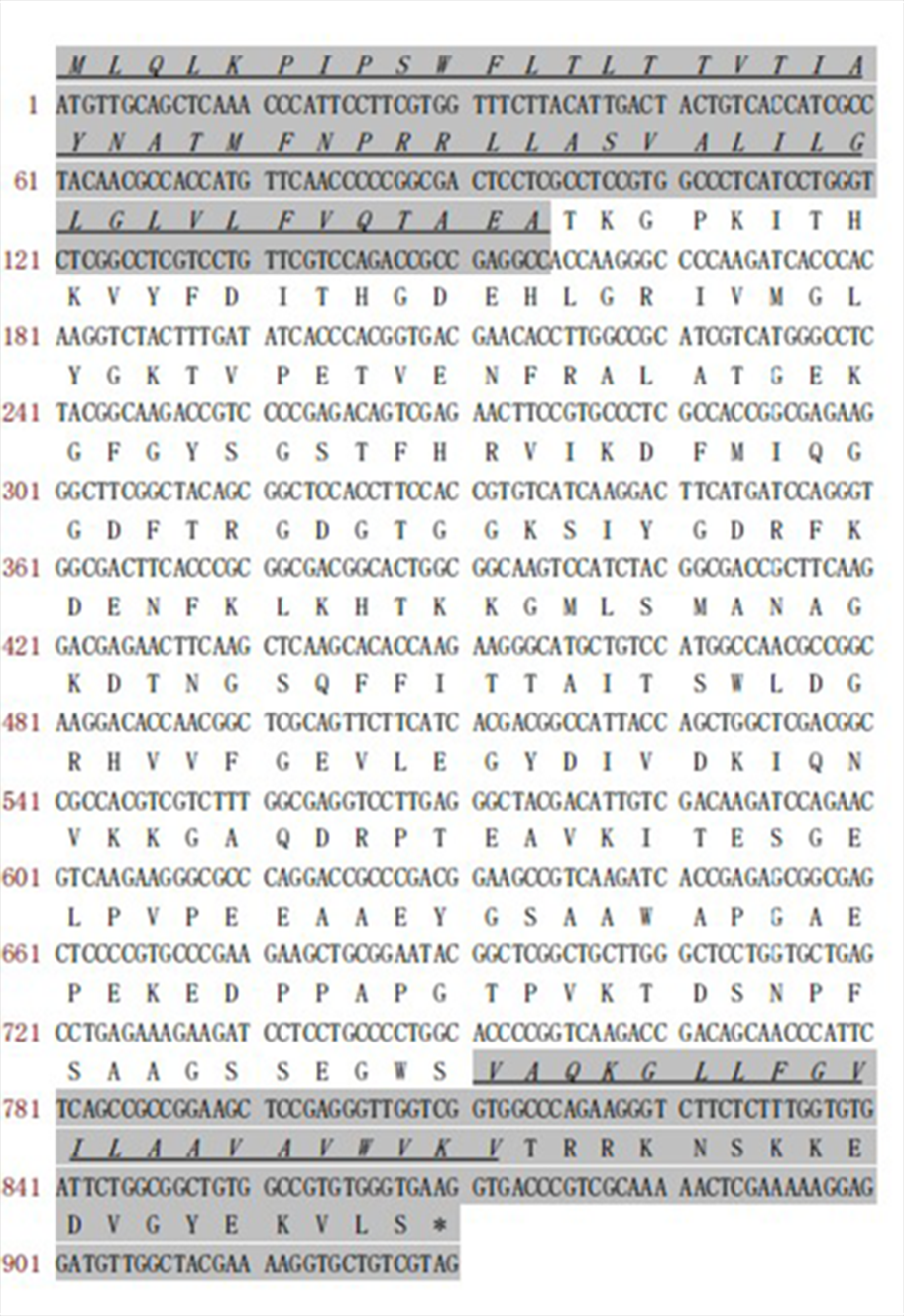

Supplement: kuae017_Supplemental_Figures [file kuae017_supplemental_figures.zip › Fig S1.tif]

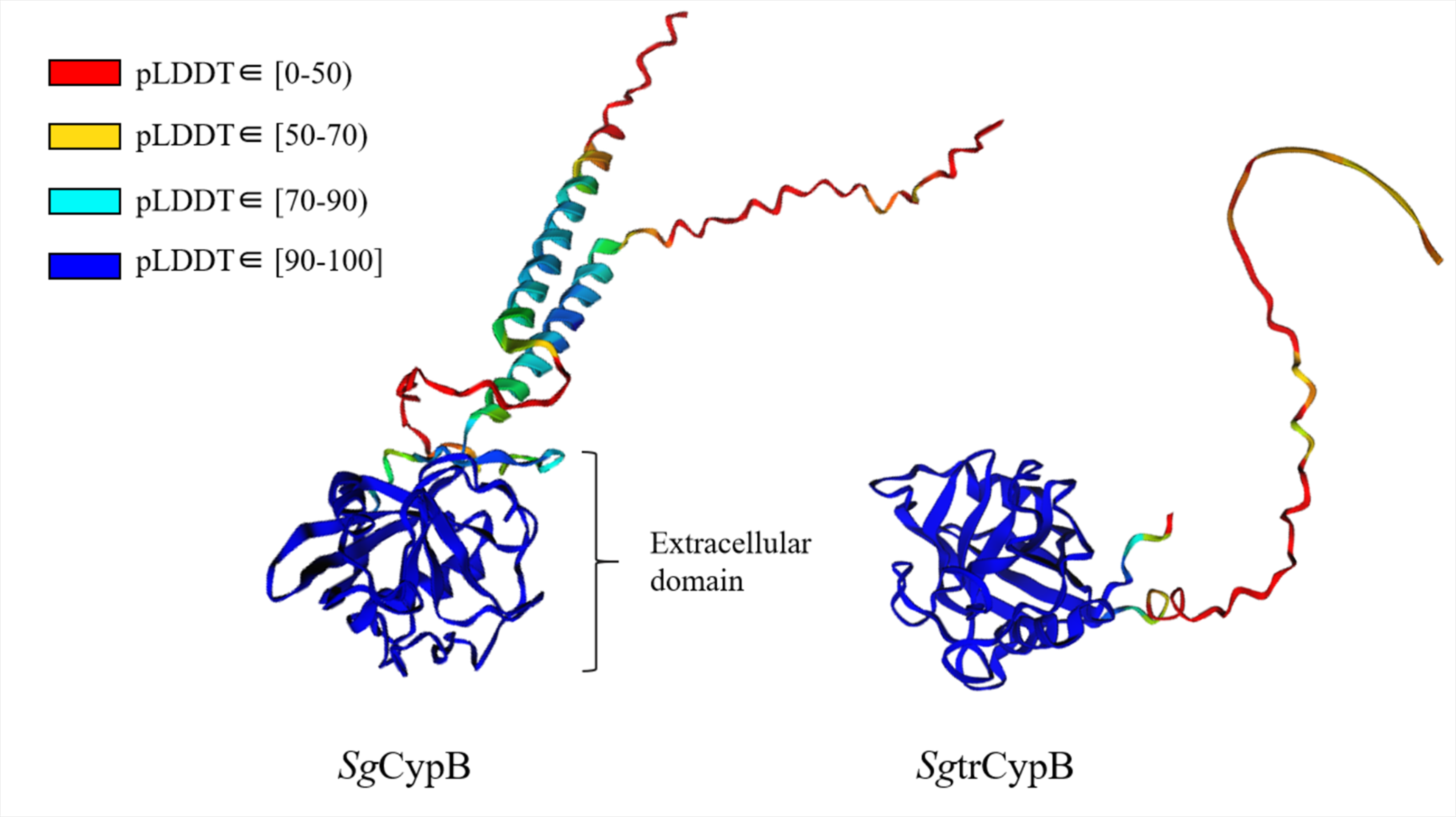

Supplement: kuae017_Supplemental_Figures [file kuae017_supplemental_figures.zip › Fig S2.tif]
